# “Take a walk in someone else’s shoes”: the role of participatory arts for health research development and training

**DOI:** 10.1186/s40900-023-00441-6

**Published:** 2023-06-08

**Authors:** Stephanie Gillibrand, Paul Hine, Rob Conyers, Jason Gravestock, Cole Walsh, Aneela McAvoy, Caroline Sanders

**Affiliations:** 1 University of Manchester, Manchester, UK; 2 Made by Mortals, Audenshaw, UK; 3 Tameside and Glossop Integrated Care NHS Foundation Trust, Ashton-under-Lyne, UK; 4 Independent (Public Contributor), Greater Manchester, UK; 5 Applied Research Collaboration for Greater Manchester, Manchester, UK

**Keywords:** Participatory arts, Theatre, Co-production, Lived experience, Marginalised communities

## Abstract

Participatory arts are increasingly recognised as a valuable and accessible mechanism for giving a voice to the experiences of individuals’ health and healthcare. In recent years, there has been a move towards embedding participatory arts-based models into public engagement processes. Here, we contribute to the existing literature on the use of participatory arts-based approaches and their role in health research and healthcare practise, focusing on two interlinked approaches, the creation of personas and storytelling. We draw on two recent projects which have utilised these approaches to inform subsequent healthcare research and as a professional training tool to improve patient experience in a healthcare setting. We add to emerging literature to outline the benefits of these approaches in supporting research and training in healthcare settings, with a focus towards the co-produced foundations of these approaches. We demonstrate how such approaches can be utilised to capture different forms of voices, experiences and perspectives to help inform healthcare research and training, rooted in the lived experience of individuals who are directly involved in the creative process of developing personas via storytelling. These approaches challenge the listener to “walk in someone else’s shoes”, using their own homes and lives as a theatrical set in which to envisage someone else’s story, involving the listener in the creative process through (re)imagining the stories and experiences of the characters. Greater use of immersive, co-produced participatory art-based approaches should be used in PPIE to inform research and training in healthcare settings as a means of centring those with lived experience through co-production. Involving those with lived experience, particularly from groups who are traditionally excluded from research, via a process which is based on co-creation and co-production, reorientates the researcher-participant dynamic to fully centre those involved in the research at the heart of the tools used to guide health and healthcare research. In this way, it may also aid in trust and relationship building between institutions and communities in a way which is focused around positive, creative methods to aid health research and healthcare processes. Such approaches may help to break down barriers between academic institutions, healthcare sites and communities.

**Supplementary Information:**

The online version contains supplementary material available at 10.1186/s40900-023-00441-6.

## Abstract (plain language summary)

This article describes how storytelling and the creation of personas, as two forms of participatory arts-based approaches, may be used to inform health research and healthcare practise, including training of healthcare professionals. We draw on two recent projects which have been created with members of diverse local communities, detailing how creative methods can be used in this way. This model of creating art with communities and people with lived experience of health conditions enables ownership of these processes and encourages participants and users to ‘take a walk in someone else’s shoes’. We suggest that using such approaches helps to further break down barriers between academic institutions, healthcare sites and communities and may boost trust between different stakeholders. Greater use of immersive, co-created participatory arts-based approaches can be used to inform research and training in healthcare settings as a means of centring those with lived experience through co-production. Involving those with lived experience, particularly from groups who are traditionally excluded from research, helps to shift power dynamics, and, in this way, may aid in trust and relationship building between institutions and communities in a way that encourages empathy generated from creative methods to aid health research and healthcare processes.

**Supplementary Information:**

The online version contains supplementary material available at 10.1186/s40900-023-00441-6.

## Background

Arts-based approaches are founded on ideals of expression, creativity and story-telling. They are particularly important for conveying complex social and inter-personal interactions, self- identity, and sentiment. In this vein, the arts are increasingly recognised as a valuable and accessible mechanism for giving voice to the experiences of individuals around health and healthcare. The role of participatory arts in delivering healthcare interventions has also proliferated [1, 2], acknowledging the benefits of such initiatives [3–7]. Public, patient involvement and engagement (PPIE) is recognised within academic best-practise as a crucial basis for ensuring research and services meet the needs of end users, and is seen as central to designing and delivering better healthcare interventions and better healthcare research [8–12]. In recent years, there has been a move towards embedding participatory arts-based models into PPIE, including the use of visual arts methods to express patient’s experiences of health [13, 14], creating textile artwork to invoke discussions around the meaning of quality in healthcare [15], and the use of analogies, props and storytelling to generate ideas on improving patient experience [16].

Here, we contribute to the existing literature on the use of participatory arts-based approaches and their role in health research and healthcare practise. We draw on two recent projects which have utilised two co-produced participatory arts-based approaches: the creation of personas and storytelling to inform subsequent healthcare research and as a professional training tool to improve patient experience in a healthcare setting. The first project was undertaken as part of the response to the covid-19 pandemic, in particular the covid-19 vaccination drive which begun national rollout in December 2020, providing insights on attitudes to the covid-19 vaccine amongst young adults in Greater Manchester in the lead up to this group being offered the vaccine. The second project describes training undertaken with health and social care staff in a hospital-setting in Greater Manchester, specifically with the focus of improving services for marginalised and under-served groups. We add to emerging literature to outline the benefits of co-produced, creative methods in approaching research and training in healthcare settings. We suggest that using such approaches helps to further break down barriers between academic institutions, healthcare sites and communities.

Arts-based approaches (e.g. role-playing, story-telling etc.) that centre compassion and empathy have long since been championed in the training of medical professionals and medical students to emulate patient experience [17–19], broadly deemed to be successful in instigating empathic responses and connection to the experiences of patients [20–23]. These are often delivered via the use of simulation-based education. For example, training student nurses about the experiences of those with sensory impairment [24], to further understanding of the daily life of those living with mental illness [25], and to simulate experiences of having melanoma [26]. Less frequently are these co-produced or meaningfully involve services users/patients directly in the creative process, in which the “role of creativity” may be overlooked [27]. The processes, methods, and materials used to develop and create simulation-based learning tools, including the extent to which patients and participants are involved in the design of such tools, is less clear.

The National Institute for Health Research (NIHR) [28] identifies five key principles of co-production in health research: the sharing of power, including all perspectives and skills, respecting and valuing the knowledge of everyone working on the research, reciprocity, and building and maintaining relationships. Areas of healthcare research have previously championed co-production, through co-produced research with families and children [29], with older individuals [30, 31] and in clinical settings [32]. Participatory arts-based approaches may be particularly well suited to forms of engagement which enable the championing of co-creation or co-production [33]. Arts-based approaches offer innovative ways of tangibly enacting co-production through the shared creation of performance art and other forms of art. Creating such pieces requires team working, relationship building, skills-sharing, communication and listening, creativity, and practical/logistical task-sharing.

The creation of art requires a form of power-sharing in the creative process which champions a diversity of skills and ideas derived from experiential knowledge, and requires an equal environment for those ideas to be actioned in the creative process in order for the creation of art to be successful [34]. Here, the act of ‘creation’ itself requires the type of collaborative skills that underpin co-production philosophies, demanding co-production in practice. This may be especially meaningful for marginalised groups, in which participatory art has been shown to positively impact those with mental health conditions, enhancing the feeling of being connected with others, building a stronger sense of identity and fostering positive feelings including hope and empowerment amongst participants [2]. Similarly, for young adults and women living in deprived areas, participatory and arts-based approaches may enhance empowerment and self-confidence [35, 36].

Two such participatory arts-based approach include the use of personas and storytelling. The creation of personas has been discussed elsewhere, within the creation of an IT-based tool for the management of hypertension [37], in the design of a mental health intervention [38] and to capture staff and stroke survivors experiences of strokes, health services and the post-stroke recovery [39] in which key features of the persona include: names, demographic details, personal characteristics including personality, interests, needs, motivations, background stories etc. [40]. Similarly, storytelling has been used to aid in the dissemination and sharing of learning and knowledge, to progress shared decision-making in healthcare and increase awareness in patients’ health-related experiences [41]. Whilst these approaches may have been championed, they do not explore the creation process itself, nor the role that both the production process and utilisation of personas has to play in re-imagining the role that such an approach can play in uplifting the voices of marginalised groups in applied examples of training and to inform research.

The examples discussed in this article use derived audio clips that have been co-produced by those with lived experience of health conditions and from diverse social backgrounds, detailing the process of their creation and utilisation in other settings. We refer below to ‘participants’ in a number of different contexts: participants involved in the creation of the *Hidden* episodes; participants involved in the workshop (in which there was some overlap between those involved in the creation of the *Hidden* episodes); and participants involved in the hospital training exercise, which involved members of staff at Tameside hospital.

## ‘Hidden’ episodes

‘Hidden’ is a series of audio-podcast episodes developed by local Greater Manchester charity, Made by Mortals. The charity creates immersive podcasts, films and music theatre shows with local communities and groups. The process puts people with lived experience into a place of leadership and expertise to share, create and gather insights from. ‘Hidden’ is a flagship series of audio experiences that is coproduced by groups and communities who are typically under-represented in research, using their imaginations and lived experience to derive and create characters and scenarios. These groups are brought together through engagement with the voluntary, community and social enterprise (VCSE) sector who work closely with Made By Mortals, including theatre companies, local charities and social prescribers. The episodes described below (Raven’s, Yousef’s and Richard’s) were used in the examples discussed in the following sections. Around 15 min in length, the episodes include characters of varying ages, with a mix of older and younger characters, with various health conditions and identity traits, from a range of socio-economic backgrounds, designed to embody qualities, experiences and stories shared by members of the group who have created each episode. The episodes explore the characters in the lead up to a moment of crisis or transition and challenges people to think about how that character could be supported in that moment. Participants are offered a shopping voucher as a “thank you” for their time.

## The process of creating the *Hidden* episodes

### Step 1: The creation of the characters

Groups and communities are brought together to collaborate with professional writers, directors, actors and sound engineers to produce their episode.^1^ The session leaders initiate a discussion with participants, to explore attitudes and perspectives on a particular subject area, with the participants drawing on their own experiences or knowledge around the topic to inform these discussions.

Using the insights from these discussions, characters are developed using drama and creative writing exercises undertaken with the group. The characters created are “fully rounded”, including, but not limited to: what they wear; what food they like; their family and/or friends; their hobbies; their home; their day-to-day life. After the character is created, the character is placed in a number of different scenarios and in a difficult situation, in a moment of crisis or transition. The group discuss the character in these scenarios: how the character feels, and the impact of certain experiences on the character. The initial discussions around the topic area and the ideas from the scenario setting generates the basis of the storyline of each episode.

### Step 2: creating the podcast

From the character creation session, professional writers (who sit in on the sessions) create a one-page document outlining a story idea or episode concept. This document is shared with the group during a group meeting (to better enable a setting where the group is supported to discuss ideas) and the group is given an opportunity to make edits and changes. The writers create a draft recording script, with another round of edits offered to the group, with the option to add additional information or lines of dialogue. Each episode is recorded with the group voicing the podcast, in which the group are again given the opportunity to edit the script. Finally, the group listens to the first draft of the recording and provides feedback to add or remove parts of the script, if necessary.

Music is created for each episode, in which the participants from the group collaborate with a professional musician to re-imagine the character’s traits and situations through music. For example, for the second episode (Yousef), the group worked with a violinist to create a piece of music that the group felt represented the feeling of psychosis. To re-create this feeling, the music has multiple different layers of sound recorded within it, as if there are lots of different voices happening at the same time. Using particular techniques, the sound effects make the listener feel like these sounds/voices are coming from different places, e.g. from the left ear, the right, in front of you, behind you, adding to a sense of confusion which echoes the sentiments of the character.

### Step 3: bringing the episode to life

When the podcast is complete the group get the opportunity to co-deliver workshops using the podcast (as the examples below discuss). The group also get the opportunity to create Additional Audio, where they and other groups of people with lived experience, have the opportunity to discuss the characters reactions, thoughts and feelings to any given circumstance. Finally, the group also get the opportunity to create “The Making of”, additional audio that explores the process of making the podcast to help communicate the power of participatory arts practice to innovate within public engagement.

### The episodes

These episodes form the basis of participatory approaches used in these examples, in which the episodes are used in a flexible, adaptive way that best suit the particular context and its aims and objectives. The following examples detail how these episodes have been used as i) a PPIE tool to inform subsequent health research and ii) as a training tool in a hospital setting.

*Richard’s Story* was made by the Johnny Barlow Theatre Company (JBTC), a group supported by Made by Mortals. The participants from the JBTC are aged 18 + and have experience of mental health conditions and/or learning difficulties. The JBTC create music theatre shows, films and audio experiences to bring about positive change in health and social care, while also improving their and their communities’ wellbeing. Working together, the group used their lived experience and imaginations to create the character, Richard. The supporting audio to Richard’s story was recorded from a variety of workshops with other groups of adults from the local community, with experience of mental health illness and safeguarding issues such as the ones encountered by Richard. The story was made in April–June 2020.

*Yousef’s Story* was also made by the Johnny Barlow Theatre Company, using their lived experience and imaginations as inspiration to create the character. The supporting audio to Yousef’s story was recorded from workshops with a Tameside based group of women of South Asian heritage, who speak little or no English. These workshops were delivered with the aid of a translator. The story was made in July 2020–September 2020.

*Raven’s Story* was made by a bespoke group of participants from Tameside aged 16–25, recruited through referrals from VCSE sector and youth services. Some participants identified as being a part of the LGBTQ + community, a few specifically being non binary. In Raven’s story, Raven is 21 and non-binary. They no longer have contact with their family and live in a bedsit in Manchester. The supporting audio to Raven’s story was recorded from a variety of workshops with other young adults from Tameside. The story was made in January–March 2021.

All episodes were made during the covid-19 pandemic with people who were socially isolating. The episodes were created and recorded using video conferencing technology and equipment was resourced for participants where needed. Some participants did not have access to the internet and so participated by dialling into the sessions using their home/mobile phones, using a free phone line number. The stories were funded by The Arts Council of England and The National Lottery, with additional funding for Raven’s episode provided by The European Social Fund.

The episodes can be found online at: https://www.madebymortals.org/hidden/

#### Young adults attitudes to the covid-19 vaccination: engagement workshop

With the ongoing rollout of the covid-19 vaccination programme in the UK, there was a need to gain an understanding of young people’s attitudes to the vaccine. Researchers at the University of Manchester wanted to engage with young people to better understand attitudes to the covid-19 vaccination, and how this interacts with experiences during the pandemic more broadly. The aim of this workshop was to inform a subsequent research project that looked at wider community experiences during the pandemic and attitudes to the vaccine, and to feedback learning to the health and social care system in Greater Manchester to better inform their vaccination strategies.

In July 2021, a group of five young adults who were involved in creating Raven’s story were brought together over an online video discussion to explore these issues, using Raven’s character and story to place Raven in a new situation around the covid-19 pandemic. Participants in the session listened to the episode and were then asked to respond to the following statements and questions:

- Raven has experienced anxiety and worry around the Covid-19 pandemic. Please can you tell us what and why this might be?
- Raven’s age group are now eligible for the vaccine. What do you think Raven’s feelings and concerns are connected to the vaccine?
- Raven feels let down by decision makers around the Covid-19 pandemic and the vaccination programme. Why? What have they missed? What are they not doing?
- What and who might help Raven feel confident about taking the vaccine?

The group of young adults contemplated each of these questions in turn, from the point of view of Raven, drawing on their own experiences, and the experiences of friends and peers to inform their responses. This approach allowed for a wide variety of opinions, attitudes, thought-processes and experiences to be articulated, and subsequently built upon during the discussion. Participants shared insights that discussed the complexities of going through gender dysphoria during a global pandemic, which compounded a heightened sense of anxiety, stress and uncertainty around the covid-19 pandemic, alongside feelings of personal loss experienced by the character. As the character of Raven lives alone, family relationship dynamics were also discussed in this context, with feelings of isolation and loneliness emphasised.

Views around the vaccine were also discussed, with the need for more information about the vaccine highlighted by participants and concerns around long term side-effects raised. The need to protect vulnerable family members, and how family and friends impact the decision to get vaccinated was also raised. Notably, the points raised in the workshop reflected many topical themes of the time, including potential health policy (mandatory vaccines and vaccine passports) and side-effects associated with the vaccines (blood clots) ﻿raised in the media. This incurred discussions around the impacts of vaccine passports on young people and how that may affect Raven’s life, as well as confidence in the vaccine and questions around the vaccines’ efficacy. Discussions also dove into the complexities of emotions and attitudes on this subject matter, in which feelings of coercion surrounding vaccination were mingled with positive attitudes towards the mass vaccination drive to enable life to “get back to normal”, especially driven from discussions around Raven’s complex mental health issues. Disillusionment and a lack of trust towards decision-makers was also raised, which spoke to a sense of the wider impact on young adults’ lives, reflecting the disruption many of them had faced during the pandemic period.

During the workshop, participants talked explicitly from their own personal perspectives, ‘Raven’s perspective, and non-descriptively a mixture of both, as well as reflecting on how other young people may feel in the context of the subject matter. Observations from Raven’s point of view, and from participants’ peers, friends, and family members were drawn on. This ‘snowballing’ effect led to detailed descriptions of experiences and attitudes that were carefully considered and clearly articulated and, crucially, allowed for a set of experiences to be heard, not just within the singular remit of the participants' lives, but from the lives of those around them, to imagine the scenarios as applicable to other young adults.

The findings from this workshop were written up into a wider engagement report [42] and circulated to the Vaccination Team in Greater Manchester, to help inform their strategic vaccination response. The insights generated from this PCIE workshop also helped to centre the experiences of young adults in a subsequent piece of formal academic research, with the aim of exploring in more depth the themes raised in this workshop. The insights fed into the co-design of the qualitative research project—helping to shape and refine research questions, inform topic guides etc.—which sought to explore views towards the covid-19 vaccine amongst local communities in Greater Manchester and their experiences of the pandemic.

#### Interactive learning with healthcare professionals at Tameside and Glossop Integrated Care NHS Foundation Trust

In the summer of 2021, Made By Mortals were commissioned by Tameside and Glossop Integrated Care NHS Foundation Trust, to provide training to volunteers and staff to facilitate understanding of different people’s perspectives to improve patient experience. This work supported the Trust’s approach to quality and diversity in supporting staff to adapt their behaviours and to create an empathetic and person-centred environment in which to better support and care for patients. The session included over 50 members of staff from a range of roles at the hospital, including people that worked on reception, porters, chaplaincy, nurses, and management.

The session was introduced by the session leader with the following statement:

*Today we are going to take a walk in somebody else’s shoes. Using immersive audio from local theatre company Made By Mortals, we will meet a character (Richard/Yousef/Raven) and learn about their life. We will then answer questions to consider what a good experience would be like for (the character).*

At the start of the session participants were invited to take part in a simple story telling exercise. This facilitated an open and safe environment and began the process of connecting the participants to the makers of the character and the character itself. After listening to three episodes of ‘Hidden’ (Richards, Yousef’s and Raven’s), participants were asked to discuss in small groups and then respond to the following questions:

- How did that make you feel, professionally and personally?
- How would the character feel about going to Tameside Hospital?
- What does a good patient experience look like for [name of ‘Hidden’ character] at Tameside Hospital or a community healthcare setting?
- What can you and your team do to give the character the best experience possible?

After each question, the participants in the session were played “the Making of” insights collected from groups of people with connected lived experience to each of the characters, responding to these questions. Using the episodes as a basis, and encouraged to position themselves within those narratives, participants were  challenged to reflect on the similarities and differences between their response and the response of people with lived experience, and given space to consider if their initial response had changed. This encouraged participants in the session to use their own voice alongside the positionality of the characters in each episode. At the end of the questions, participants were challenged to make a behaviour change pledge based on their experience within the training. Examples of these pledges included: being more aware to difference, greater listening and communication, being honest and open about unconscious biases, using appropriate pronouns and being responsive to the use of pronouns (including asking people how they would like to be referred to), being open-minded and aware of individuals’ situations and experiences and centring this in interactions with patients. Feedback from participants highlighted that they felt a greater sense of belonging and community, alongside a better appreciation of inclusion, where all ideas are viewed with equal importance.^2^

The approaches utilised in this session have been mainstreamed into part of the ongoing integrated training and awareness work supported and delivered by the Patient Experience Team at Tameside Hospital. This aims to become a mainstreamed approach which further supports colleagues and volunteers across the organisation to consider the differing voices, perspectives and experiences of people accessing services. Insights from the session has supported the Trust’s approach to equality and diversity, as the work is recognised as an important basis to facilitate an improved patient experience rooted in person-centred care. It is intended that this will form the basis of delivering this type or training to further teams in the hospital (i.e. receptionist teams) to more consistently apply the awareness generated and learnt across different parts of the hospital.

## Discussion

The examples discussed here demonstrate how co-produced participatory arts-based approaches can be utilised to capture different forms of voices, experiences and perspectives to help inform healthcare research and training, founded upon the lived experience of individuals who are directly involved in the creative process. This helps to keep the characters grounded in reality and builds connection between the group and the characters.

Opposed to more traditional means of art-based training in healthcare settings, (i.e. simulation-based processes), these initiatives directly involve participants in the creative process, inducing a sense of ownership and closeness to the characters and their experiences. Here, the process of co-production itself is inherently valuable to showcase lived experiences and include the perspectives of lived experience in this way [43]. For participants with lived experience involved in the creative process of character development, it creates a sense of connectedness via the establishment of a community through the group development of a creative piece. Such processes also hold a cathartic nature for the participants, in which portraying ones’ own experiences in a creative form validates these experiences and privileges them as story to be told to the outside world by virtue of recognition of these experiences. As the episodes are then used in applied contexts, it builds on the creative development process as a potential mechanism to achieve change through awaressness building.

Facilitating the development process in a group setting enables each participant to contribute to a piece of that character, which allows the characters to be shaped by multiple individuals’ narratives with a range of lived experiences. This means the character does not reflect a singular participant but each of them, avoiding a uniform extrapolation where the character simply embodies a singular perspective based around one person’s experiences. This may be even more apparent when these scenarios and characters are then applied and used by other participants in additional contexts, in so far as the combination of voices, perspectives, narratives and experiences from multiple angles and across contexts removes the possibility of a singular voice dominating the character’s identity. In addition, utilising the characters’ experiences to explore topics alongside the participants’ own voices, allows for an inversion of the character/audience (participant) dynamic, in which participants are encouraged to internalise and empathise fully with the characters, enacting an iterative fluidity which allows for participants to incorporate their own views, experiences and perspectives into the discussion.

These approaches can play an instrumental role in the training of medical professionals, where traditional “training” methods are not sufficient to fully understand the complexities of another’s lived experience. To this end, staff members at Tameside hospital were not told that the event was specifically a “training” event, in order to encourage staff to bring their own experiences to the table. It was envisaged that this would enable staff to immerse themselves in someone else’s life in a more ‘natural’ setting, rather than seeking to gain additional “skills” in the way traditional ‘training’ events are set up to do; to develop an awareness of the challenges and barriers that people from different backgrounds experience, using their voice alongside the characters’ experiences, for staff to talk about how they can respond to this in their own professional practice. This meant that there was a more open-minded ethos around the tone of the session where participants were more amenable towards the topics of discussion. For example when a member of staff spoke about their child being non-binary, it led to a wider discussion about the general lack of knowledge and awareness of non-binary individuals’ experiences and lives, which reiterated the discussion points around thinking from others’ perspectives. As the stories within the audio podcasts are co-produced with service users themselves (those with lived experience), the ensuing discussion is more intimate as it is rooted in real-life stories, and therefore when the episodes are used in other contexts this ethos is built on.

By portraying a well-rounded, fully-developed character, participants are encouraged to engage with all aspects of the characters’ life, including how the characters’ experience their life in general, alongside positive and negative aspects of their life, which in turn enables a better sense of understanding of how the characters navigate their lives and how they may overcome specific barriers and challenges. By having time to actively listen, think, engage, and then discuss the changes they (as healthcare professionals) could enact, the aim was to develop a basis of understanding founded upon iterative learning, that can inform each individual’s future practice. As such, the actions and pledges from the main discussion points were focused around the individual participants themselves, but some also had wider relevance to the systems and structures that run the hospital. For instance, the pledges included personal commitments, such as challenging stigma, using the correct pronouns etc., and more system-based ones including leading the way for more inclusive working, identifying what structures may help and hinder this and targeting efforts accordingly, and leading workforce development initiatives that promote principles of inclusion.

This offers wider opportunities for the role of “participation” in training in healthcare settings, moving away from more traditional methods where participation is limited to either a consumer or benefactor, rather than engaging directly in the creative process [44] where dichotomies of trainer/trainee are constructed [45]. For example, traditional simulation-based learning techniques tend to utilise pre-existing patient-based scenarios to enact solution-based learning, in which the interaction is limited to participation (in the simulation), observation and debriefing [24, 46]. Other methods such as vignettes have been used to stimulate discussion for interview-based research, however it has been found that using vignettes in this context has incurred a lack of understanding by researchers of the use of vignette characters to facilitate discussion at the expense of allowing participants to share their own experiences [47]. Here, the process of co-production itself is inherently valuable to showcase lived experience and include the perspectives of lived experience in this way [48]. In contrast, the approaches outlined above invoke a model which requires deeper engagement in the creative process, by requiring the participants to have a contributing voice themselves in both the development process and applied contexts, whereby the dichotomy of participant/character is purposefully blurred.

The development process of the episodes and the contexts in which they are applied also provides a layer of confidentiality and anonymity, where the setup is flexible enough that it is possible for participants to discuss potentially sensitive issues or personal experiences without necessarily revealing whether the insights discussed are drawn from their own personal experiences, or imagined through the lens of a character. This enables participants from the groups to share ideas or personal experiences via the character, without them feeling like they have revealed their personal experiences in an exposing manner. This meant participants could project themselves onto the characters, sharing their experiences in a controlled, safe manner, by disclosing the level of detail they felt comfortable with. For instance, in the vaccination engagement workshops, participants would often switch between perspectives when discussing the topics. In this way, this approach to PPIE allows for the discussion of sensitive or contentious subject matters (such as the covid-19 vaccination) to be broached with a degree of separation between the participants and the area of discussion, which enables participants to feel at ease discussing sensitive subject areas.

Co-creation of the episodes also seeks to address power disparities in the co-production process, in which participants have decision-making power over the scope and overall direction of the ‘Hidden’ episodes. Often the scope and problem framing of a project is pre-defined [49], meaning there is not complete ownership from communities and it is hard for decision-making autonomy to be truly handed over to communities themselves. That being said, it cannot be claimed that power is completed devolved, as funding and resource capacity is still located within the realms of the institutions of partnering team members. However, this process supports people with lived experience into a place of leadership and expertise to share, create and offer insight which enables health researchers and healthcare practitioners to hear the voices of communities and to subsequently support decision-making mechanisms which champion lived experience. In addition, using the episodes in applied contexts which directly immerse and involve the participants, blurs the lines between ‘performer’ & ‘audience’, ‘participant’ & ‘researcher’, ‘service user’ & ‘service provider’ ‘trainer & trainee’, to disrupt traditional power structures in these settings. In this way, such approaches epitomise the underpinning values of meaningful co-production techniques, in which power-sharing and providing communities with great control over the research process is a key proponent of co-production [50, 51]. The use of storytelling and personas follow existing arts-based methods in this space, including a virtual photo exhibition on mental health during the covid-19 pandemic,^3^ an inter-disciplinary co-produced “collaborative poetics” approach to research [52], in which these approaches are increasingly being utilised in health and healthcare relevant settings. The work presented here presents further opportunities for inclusive, co-produced participatory-arts based approaches, centring the experiences of marginalised group to better support the improvement of services in healthcare setting and to convey experiences and perspectives across the public health fora more broadly.

The benefits of participatory co-produced approaches have been well-documented, including individual level empowerment [53] and community-level empowerment [54]. Participatory arts-based approaches may also act as a tool to improve the health of participants themselves, through sharing experiences and stories within a team setting, meeting like-minded people from similar communities. This is supported by existing research in this field, which documents the wellbeing and emotional health benefits of participatory arts-based approaches for those involved [55–57]. In this way, participatory approaches also offer innovative avenues of engagement that may resonate better with certain groups or communities, as a method that sits outside of ‘traditional’ engagement processes, such as surveys, focus groups and even social media tools (polls etc.). The use of participatory approaches also alleviates pressure from marginalised groups to be able to recount their experiences and stories of their lives in a perfect, precise manner in a one-off setting. Creating characters and stories allows the nuances and complexities of individuals’ lived experiences to be conveyed in a natural way that captures the varying, imperfect aspects of peoples’ lives that can be hard to capture through more traditional methods. Such approaches also facilitates breaking down barriers between the communities and research and academic institutions, healthcare sites and settings, through which engaging in participatory-arts is seen to foster a sense of community, civic participation and strengthened relationships, whereby participatory activities are seen as “safe spaces” built around principles of equality and openness [58]. This may also foster increased trust between marginalised communities and health and social care providers, where distrust towards mainstream services may be more distinct amongst marginalised groups.

Scholars have previously advocated for co-created arts-based approaches that break down barriers between communities and patients, academics and healthcare workers [59, 60], as well as activities that tap into empathetic responses, such as role-playing and theatre-based activities, which is identified as beneficial to both patients and healthcare workers themselves [61]. Participatory theatre approaches are used to create safe, innovative environments in which creativity and imagination can be used to explore complex topics, in a way that is outside of traditional story-telling methods, i.e. verbally or in a written format [62] to promote awareness and stimulate conversation around these topics [63], to enact reflective and social critical dialogue between community members and partners. Similarly, participatory arts have been used in the research of sensitive topics, such as mental health, in culturally sensitive conditions with minority populations, to illicit a safe-space environment and to help break down communication barriers in the discussion of sensitive topics [61], recognising the need for ‘culturally appropriate’ approaches for different groups. This is particularly pertinent in the example of PPIE with younger adults, as a group who are traditionally under-represented in academic research, both in research priorities and the research itself. It also reiterates the importance of using culturally appropriate approaches for different groups, on the intersectional basis of demographics and other characteristics (such as those typically excluded from research) [64]. Involving young adults in this innovative way addresses the need to “to devise engaging, relevant ways of working with young people with meaning and purpose, and to avoid tokenism” [61], and has additional benefits of empowerment, self-confidence and the opportunity to develop new skills (ibid, pp. 3–4).

All withstanding, the methods discussed here, and participatory-based methods more generally, cannot be done without the appropriate institutional infrastructure and sufficient resource base, which serves as the backbone to generate the different skills and partnerships needed for these approaches to be successful *in the right way*. For instance, it was important to have buy-in from key staff members for the hospital session to dedicate staff time, including senior staff to participate in the session (the session included those from Bands 3- 8), to ensure that senior leaders were involved and able to lead on taking forward the points raised in the session. In addition, the PPIE was conducted online due to the covid-19 pandemic, which allowed for an immersive experience in a familiar and accessible setting i.e. in the participants’ own homes. Whilst this does not avert barriers of access for people who are digitally excluded, it provides an element of flexibility for participants who do not have to travel to a specific venue to participate, in which time and money constraints may be limiting factors in this regard. It may also be more accessible for those with mental health issues, in which an online setting that can be attended from a familiar and safe setting may reduce barriers to attending in person. Indeed, remote methods provided new opportunities for PPIE with marginalised groups during the pandemic, although issues prevail around the insect of digital access and poverty and inequality [65]. To that effect, funders and institutions must continue to prioritise and recognise the value of these approaches through maintaining commitment through appropriate investment and resource in order to undertake participatory-arts based approaches in an inclusive manner.

## Conclusion

The examples discussed here as examples of co-produced participatory-art based approaches-storytelling and the creation of personas - form the basis of PPIE for health research and training in a healthcare training. The approaches discussed here invoke a strong emotive response for participants who utilise the resources. Deep-rooted in empathy and understanding, these approaches humanises scenarios for the participants involved, making the scenarios less abstract by breaking down barriers between an abstract ‘other’ and ourselves. Empathy-based approaches are already recognised as fundamentally important in the healthcare profession, in particular for the training of medical professionals. The co-production of these approaches offers new frontiers for this type of training, rooted in the lived experience of others that invert the trainer/trainee dynamic. These approaches challenge the participants to “walk in someone else’s shoes” using their own homes and lives as a theatrical set in which to envisage the points for discussion with someone else’s story, involving participants in the creative process through (re)imagining the stories and experiences of the characters. Utilising such approaches delivers an innovative and creative approach to aid in the design of healthcare research, as well as training programmes in a healthcare setting. Elements of this type of participatory approach may be easily streamlined into PCIE/PPIE practises, to incorporate aspects of creativity, imagination and character development into PPIE approaches to enable different perspectives and points of view to be more fully considered in the PCIE process and therefore articulated into training and research.

Greater use of immersive, co-produced participatory art-based approaches should be used in PPIE and training in healthcare settings as a means of centring those with lived experience through co-production. Involving those with lived experiences, particularly from groups who are traditionally under-represented in research, via a process which is based on co-creation and co-production, re-orientates the researcher-participant dynamics to fully centre those involved in the research at the heart of the tools used to guide health and healthcare research. In this way, it may also aid in trust and relationship building between institutions and communities in a way which is focused around positive, creative methods to aid health research and healthcare processes. Centring the voices of diverse communities as the main mechanism for training helps to aid in learning, using tools from an empathetic position and story-telling to inform narrative based learning. For participants involved in creation of personas, creating something which invokes an emotive response is therefore likely to inspire feelings of empathy, which is in turn a positive motive for change. In this way, these methods can be a potential mechanism for change as the nature of the approaches inspires people to listen to the voices of others, and even challenge their own ideals to bring about change (Additional file 1).

## Supplementary Information

**Additional file 1:** GRIPP2 reporting checklists: tools to improve reporting of patient and public involvement in research.

## Data Availability

Not applicable.

## Abbreviations

ARC: Applied Research Collaboration for Greater Manchester
NIHR: National Institute for Health Research
JBTC: Johnny Barlow Theatre Company
PPIE/PCIE: Public/patient/community involvement and engagement
VCSE: Voluntary, community and social enterprise sector

## Footnotes

1 Due to the covid-19 pandemic, the sessions were undertaken online, by remote video-conferencing.

2 Further details and feedback from participants are provided here https://www.madebymortals.org/person-centred-approaches/

3
https://healthinnovationmanchester.com/news/what-hopes-means-during-the-covid-19-pandemic-a-virtual-exhibition-to-mark-world-suicide-prevention-day/.
