# Supplementary material for: “Take a walk in someone else’s shoes”: the role of participatory arts for health research development and training
Source: Res Involv Engagem. 2023 Jun 8;9:40. doi: 10.1186/s40900-023-00441-6 (PMC10249567; doi:10.1186/s40900-023-00441-6)
Supplement: Supplementary file 1 — Additional file 1: GRIPP2 reporting checklists: tools to improve reporting of patient and public involvement in research. [file 40900_2023_441_MOESM1_ESM.docx]

**Appendix 1**

[GRIPP2 reporting checklists: tools to improve reporting of patient and public involvement in research](https://researchinvolvement.biomedcentral.com/articles/10.1186/s40900-017-0062-2)

| **Section and topic** | **Item** | **Reported on page No** |
| --- | --- | --- |
| 1: Aim | Report the aim of PPI in the study | 3, 5-6 |
| 2: Methods | Provide a clear description of the methods used for PPI in the study | 6-8 |
| 3: Study results | Outcomes—Report the results of PPI in the study, including both positive and negative outcomes | 9-10 |
| 4: Discussion and conclusions | Outcomes—Comment on the extent to which PPI influenced the study overall. Describe positive and negative effects | 10-13 |
| 5: Reflections/critical perspective | Comment critically on the study, reflecting on the things that went well and those that did not, so others can learn from this experience | 10-13 |
